# Supplementary material for: Exploration of a workflow for the classification and identification of co-purified protein complexes yields new structures and multiple MSP assembly states
Source: PLoS One. 2026 Jan 27;21(1):e0340207. doi: 10.1371/journal.pone.0340207 (PMC12843557; doi:10.1371/journal.pone.0340207)
Supplement: Attachment — Submitted filename: Replies to reviewers.docx [file pone.0340207.s015.docx]

Review Comments to the Author

We appreciate the time of the reviewers and the critical reading of our manuscript. The comments have strengthened the manuscript, and readability. We highlighted changes to the manuscript in red to simplify the review process.

Reviewer #1: In this manuscript, the authors of this work suggested that membrane proteins and membrane scaffolding proteins interact directly. Please give a response to the following:

1) What is the biological relevance of docking scores?

Thank you for pointing out the weakness in explaining the scoring used. We expanded our explanation for the procedure for the unsupervised model building in the main text. P.4 l.89-91 and p. 6. l.132-134. In addition, we expanded the Methods part to include a description of how we arrived at the ModelAngelo model, p.15 l. 382-390. In short, we are mostly relied on the E-value which is the probability of false positive sequence values for the whole modeled segment/fragment. We included now the output for ModelAngelo and the corresponding values for each fragment in the supporting information (Tables 3-5). As for the finalized model that we generated manually, we first fitted in and then adjusted sidechains etc. The final refinement, meaning the final adjustment of sidechain angles etc. was done with established programs (Phenix, Servalcat), which produced the parameters in Table 1. Therefore, the quality of the initial docking of the model into the cryoEM map is not as important anymore.

2) The molecular docking results are intriguing, but is there any experimental validation? This necessitates investigations (such as binding assays) to validate any interactions with these proteins.

We appreciate the comment and hope to provide some clarification. For ACRB and ARNC only protein models (pdbs) were refined into the cryoEM densities. We have observed low resolution densities representing lipids, but they were far from usable for modelling. For the putative interactions of the membrane protein with the MSP. We tried to reconstruct the cryoEM map for the MSP. However, we never obtained good results that we could use to pinpoint interactions between the membrane protein and the MSP density. Therefore, we refrained from speculating which MSP residues are interacting with the respective membrane protein.

For BO3, we obtained maps that have discernable densities in the position of lipids. Further, we identified densities for the heme groups and well as the ions. The quality of these two groups of non-protein densities was different. For the heme groups the density was well enough defined to correctly assign them. For the Cu and Zn the positioning of these ions were at positions, where we would expect them. Lipid densities are a challenge because we are not sure about their biochemical identity due to the uncertainty of the exact composition of the used soy lipids. So, we refrained from modeling lipids, but referred to findings of others, which seems consistent. However, we modelled and refined the hemes and ions, which is why we used Servalcat. This is the reason why we supplied the fitted lipid densities as supporting information and not deposited these lipids in our model.

3) Please provide the gel's figure in the manuscript, but do not provide it as supplemental information.

The gel image is now new Figure 1, we supplied the gel as a separate image in the supplemental.

Reviewer #2: The article “In silico classification and identification co-purified protein complexes yield new structures and multiple MSP assembly states” by Qingyang Zhang, Abhinandan Venkatesha Murthy, and Carsten Mim present a study of the resolution of membrane proteins via cryoEM after the use of nanodiscs and a rather limited purification.

The article basically presents two aspects:

the methodological aspects in getting the models

observations of the interactions of nanodisc scaffolding proteins with the purified proteins.

The first aspect is put forward, including in the title. It is unclear to this reviewer what justifies this choice, the novelty being limited from what is described in the text.

For example, the paper is written in a way that may lead the reader to think that identifying proteins via cryoEM is new, as the authors have a section titled “Unsupervised model building of cryoEM maps can identify proteins”. This is however the results of the paper presenting ModelAngelo. It is unclear if the authors have built further on that because the ways proteins are recognised is not detailed:

lines 120-121 the authors state “Nevertheless, ModelAngelo identified the protein as Undecaprenyl-phosphate 4-deoxy-4- formamido-L-arabinose transferase (ARNC)” but no details are given and ModelAngelo is not even mentioned in the Methods section The authors should probably consider changing their wording, including in the title to remove the apparent claims for novelty that are not justified. The observations around the structure of nanodiscs and interaction of scaffold proteins with proteins of interest would probably deserve to be more at the center of the attention of the readers.

Thank you for the comment and helping us to avoid any confusion about our contribution.

We modified the title to make it clearer that we are proposing a new workflow. To emphasize this, we are including the information that we are using publicly available software already in the abstract (p.1 l.17-18). We point out in the introduction that other packages exist (p.3 l.58-59) and our reasoning to use ModelAngelo (p.4 l.89-91 and p. 5 l.109). We also expanded on our criteria to assess the hits (p.5 l.126-128) and supplied the output from ModelAngelo in the supporting information (Tables 3-5). We added a separate section in the Methods (p. 15 l.382-390) on the unsupervised model building.

We do believe that our workflow is novel in the sense that we do not require any *a priori* knowledge about the maps we reconstructed. The ‘build-and-retrieve’ workflow, requires model building and then repicking or mass spectrometry. In fact, our mass spectrometry data provided so many hits, that we still had no idea about the identity of the proteins, which stalled the project. We think that our proof-of-concept will be useful for other scientist that may not have access to a mass spectrometry facility or would like a ‘quick-and-dirty’ overview what may be present in a mixture. The report introducing ModelAngelo demonstrated that it is possible to identify proteins *de novo*, yet we wanted to test it in a new setting and a specific workflow dedicated to identifying new proteins.

As for the second part regarding the nanodisc assemblies, this was a surprising finding and is not very well documented. It is an emerging realization that membrane scaffold proteins may interact with membrane proteins. Here, we present evidence that this happens in 3 different proteins and that this has an influence on the nanodisc itself. If this is universal needs to be shown.

In conclusion, we believe that both components are a compelling aspect of this study, even if they are somewhat unrelated.
