# Supplementary material for: Exploration of a workflow for the classification and identification of co-purified protein complexes yields new structures and multiple MSP assembly states
Source: PLoS One. 2026 Jan 27;21(1):e0340207. doi: 10.1371/journal.pone.0340207 (PMC12843557; doi:10.1371/journal.pone.0340207)
Supplement: Attachment — Submitted filename: Response_to_reviewers.docx [file pone.0340207.s016.docx]

We thank Reviewer #3 for the time to comment on the manuscript. The suggestions made the manuscript stronger. Below we respond the each point.

1. The authors mixed up “ubiquitin” with “ubiquinone” throughout the manuscript. This is a major scientific error. Ubiquitin is a protein and ubiquinone, or coenzyme Q is a ligand.

We thank the author for making us aware of this major mistake. We replaced all instances of "ubiquitin"

2. In the abstract the author claims that the approach “we only require electron micrographs and a computer for unsupervised model building and protein identification…”. However, identifications are ultimately verified by MS so this claim is a bit too strong in my opinion.

We appreciate the suggestion and changed the text accordingly. We emphasized the tentative nature of our approach (l.19-20) and made clear that we used MS to validate our workflow. We also added that we used MS in the introduction and the discussion (l. 66, l. 160).

3. The approach seems work well for <4 Å maps and proteomes with good coverage in ModelAngelo/HMMer. Please discuss failure modes (lower resolution, low abundance targets, heavy PTMs/unknown ligands, etc) and when other evidence like MS becomes essential.

We agree that our approach is promising but has the pitfalls pointed out by the reviewer. We noted that the ARNC was assigned correctly, however due to the low resolution the connectivity and some sidechain assignments are wrong (l. 155-157). ARNC is a relatively small protein, but there were enough fragments to identify the protein. For the first revision, we hinted at these problems (l. 278-280). We followed the reviewer’s suggestion and added more disclaimers (l. 283-288). This includes our case where we saw non-protein densities in the BO3 structure but could not assign them.

4. The authors claim that “one MSP2N2 molecule is enough to form a nanodisc”. This seems to be based only on the diameter estimates and the appearance of “rungs” in the belt density. Please either provide stronger evidence, soften the claim or discuss alternative models at minimum. Similarly, I think the evidence for “direct interactions between the embedded protein and the scaffolding protein” is not strong enough. Without an atomic MSP model or orthogonal evidence, “direct interaction” should be framed as a hypothesis.

We agree with the reviewer that our writing was too enthusiastic. We formulated our findings more neutral. We changed the heading of the paragraph (l. 176-178). We also made it clearer that it is our interpretation that density represents MSP(l. 194-197, legend Figure 4). See also point 6.

5. For model building and confidence, it would be helpful to add a compact, target-by-target summary of ModelAngelo/HMMER scores (E-value, bit score, bias) and a clear rule set for accepting IDs. Where models are guided by AlphaFold or PDB homologs, explicitly label those regions as guided rather than unsupervised. A residue-wise confidence plot (e.g., per-residue map CC or Q-scores) for the final models would be valuable.

We are thankful for the comment and the opportunity to clarify. We added a new table (S6 Table) that lists all targets only. We have no clear cut off for the E-value or any of the other scores. We could imagine that these thresholds are variable, when comparing two isoforms or more broadly two protein family members. Visual inspection of key residue density would be more helpful. The unsupervised model building via ModelAngelo served as an identification tool only. The models were either built based on a pdb template (BO3 and ACRB) or an AlphaFold model. We clarified that in the text (l. 140-141, 147 and 153) and in the figure legend for Figure 3 (l. 172-174) and the Methods part. To address the model quality, we expanded the S1 Table with a section only dealing with the model quality, including the Q score, Atom inclusion and the Map-Model CC. The submitted validation reports contain per sidechain metrics for each of the models. For transparency, we are uploading the original movies to EMPIAR (deposition ID 47487413).

6. The reported diameters vs. a 165 Å “ideal” MSP2N2 disc are central to the conclusions. I think the analysis is not enough here. Please provide a quantitative analysis like distributions from 2D classes and/or map segmentations, measurement methodology, uncertainty estimates, and whether per-view anisotropy or masking could bias diameters measurements.

The reviewer is right that a more precise description would be helpful. We estimated the width of the nanodisc manually. We added this information to the main text (l. 184), figure (l. 211-212) and methods section (l. 434-435). We believe it is more reliable than measuring 2D classes, where tilted projections can distort the distance measurements. The reconstruction mask did encompass the whole nanodisc. We added references describing the size of empty nanodisc. We also searched for cryo-EM maps that encapsulated membrane proteins with MSP2N2. In three cases, the nanodisc was between 10-12nm and therefore smaller than an empty MSP2N2 nanodisc. This finding supports the idea that nanodiscs assembly may be influenced by membrane proteins. A recent paper showed that nanodiscs are dynamic in solution, to the point of near disintegration. We added these references into the main text.

Minor issues

1. I have noticed several grammar issues and typos. I suggest the authors should carefully proofread the manuscript again. For example,

(1) Line 132. An extra “a” in “We did not supply an individual a sequence, …”.

(2) Line 256. “Solving the structures native proteins…” should be “Solving the structures of native proteins…”

(3) Line 342. A missing “and” in “The pooled fractions were concentrated subjected to SEC…”

(4) Line 374. “…, resulting poorly resolved…” should be “… resulting in poorly resolved…”

(5) Line 397. “BO03” should be “BO3”.

We thank the reviewer for noting the errors and understand it impacts the reading. We addressed these and other errors throughout the manuscript.

2. Also, some nomenclatures should be consistent throughout. For example, mixed usages of (1) cryoEM/cryo-EM, E. coli (with space)/E.coli (without space), And explain what are hZANC (human Zinc activated ion channel?) and MSP2N2 (membrane-scaffold-protein 2N2?).

Again, we apologize for the inconsistencies and read the manuscript carefully to correct them.
